# The utility of methylmalonic acid, methylcitrate acid, and homocysteine in dried blood spots for therapeutic monitoring of three inherited metabolic diseases

**DOI:** 10.3389/fnut.2024.1414681

**Published:** 2024-06-20

**Authors:** Yi Liu, Xue Ma, Lulu Kang, Ying Jin, Mengqiu Li, Jinqing Song, Haixia Li, Yongtong Cao, Yanling Yang

**Affiliations:** ^1^Department of Clinical Laboratory, China-Japan Friendship Hospital, Beijing, China; ^2^Department of Pediatrics, Peking University First Hospital, Beijing, China; ^3^Department of Pediatrics, The First Affiliated Hospital of Zhengzhou University, Zhengzhou, China; ^4^Department of Clinical Laboratory, Peking University First Hospital, Beijing, China

**Keywords:** homocysteine, homocystinemia, inherited metabolic disease, metabolic evaluation, methylcitric acid, methylmalonic acid, methylmalonic acidemia, propionic acidemia

## Abstract

**Backgroud:**

Routine metabolic assessments for methylmalonic acidemia (MMA), propionic acidemia (PA), and homocysteinemia involve detecting metabolites in dried blood spots (DBS) and analyzing specific biomarkers in serum and urine. This study aimed to establish a liquid chromatography–tandem mass spectrometry (LC–MS/MS) method for the simultaneous detection of three specific biomarkers (methylmalonic acid, methylcitric acid, and homocysteine) in DBS, as well as to appraise the applicability of these three DBS metabolites in monitoring patients with MMA, PA, and homocysteinemia during follow-up.

**Methods:**

A total of 140 healthy controls and 228 participants were enrolled, including 205 patients with MMA, 17 patients with PA, and 6 patients with homocysteinemia. Clinical data and DBS samples were collected during follow-up visits.

**Results:**

The reference ranges (25th–95th percentile) for DBS methylmalonic acid, methylcitric acid, and homocysteine were estimated as 0.04–1.02 μmol/L, 0.02–0.27 μmol/L and 1.05–8.22 μmol/L, respectively. Following treatment, some patients achieved normal metabolite concentrations, but the majority still exhibited characteristic biochemical patterns. The concentrations of methylmalonic acid, methylcitric acid, and homocysteine in DBS showed positive correlations with urine methylmalonic acid (*r* = 0.849, *p* < 0.001), urine methylcitric acid (*r* = 0.693, *p* < 0.001), and serum homocysteine (*r* = 0.721, *p* < 0.001) concentrations, respectively. Additionally, higher levels of DBS methylmalonic acid and methylcitric acid may be associated with increased cumulative complication scores.

**Conclusion:**

The LC–MS/MS method established in this study reliably detects methylmalonic acid, methylcitric acid, and homocysteine in DBS. These three DBS metabolites can be valuable for monitoring patients with MMA, PA, and homocysteinemia during follow-up. Further investigation is required to determine the significance of these DBS biomarkers in assessing disease burden over time.

## 1 Introduction

Methylmalonic acidemia (MMA), propionic acidemia (PA), and homocysteinemia represent rare yet potentially serious inherited metabolic disorders stemming from a various congenital errors in cobalamin (cbl), propionate, or homocysteine metabolism (1–4), which were detailed in Table 1. MMA and PA are relatively common organic acidemias, with MMA further categorized into methylmalonic acidemia combined with homocysteinemia (combined MMA) and isolated methylmalonic acidemia (isolated MMA) based on the presence or absence of elevated homocysteine (1, 5). Homocysteinemia encompasses a spectrum of rare conditions such as cystathionine β-synthase (CBS) deficiency and disorders related to homocysteine remethylation (6, 7). The clinical manifestations of these inherited metabolic disorders span from mild late-onset forms to severe early-onset presentations (8, 9). In clinical practice, the biochemical diagnosis of the above disorders is typically established through the assessment of screening indicators (propionylcarnitine, methionine, and their ratios) in dried blood spots (DBS), as well as specific markers (such as methylmalonic acid, methylcitric acid, and homocysteine) in urine and serum (3, 10). Ding et al. (11) analyzed the data of 85 patients with late-onset MMA and found that the time from onset to diagnosis was an independent risk factor for poor outcomes, such as movement disorders, recurrent seizures, intellectual disability, chronic renal failure, and progressive pulmonary hypertension. In another study that recruited 365 patients with mut-type MMA (12), patients who underwent newborn screening (NGS) had a higher health rate and lower rates of death and neurological sequelae than those with disease onset in non-NBS group. Therefore, timely identification and appropriate intervention have the potential to improve clinical outcomes.

**Table 1:** Expected metabolite abnormalities, causing genes and therapies of different disease groups of propionate, cobalamin and homocysteine metabolism classified by biochemical pattern.

Typical biochemical phenotype					Biochemical diagnosis		Enzyme defect	Causing gene	Conventional therapy
C3	Met	Mma	Mca	Hcy	Disease group	Subgroup			
↑	–	↑	−/↑	↑	Methylmalonic acidemia	Combined with homocysteinemia	clbC cblD cblF cblJ	*MMACHC* *MMADHC* *LMBRD1* *ABCD4*	Vitamin B12, Carnitine, Betaine
↑	–	↑	−/↑	–		Isolated	MUT cblA cblB cblD variant 2	*MMUT* *MMAA* *MMAB* *MMADHC*	Dietary management, Vitamin B12, Carnitine
↑	–	–	↑	–	Propionic acidemia		PCC	*PCCA* *PCCB*	Dietary management, Carnitine
–	↑	–	–	↑	Homocysteinemia	Classic	CBS	*CBS*	Dietary management, Vitamin B6, Betaine
–	−/↓	–	–	↑		Remethylation disorders	MTHFR cblD variant 1 cblE cblG	*MTHFR* *MMADHC* *MTRR* *MTR*	Folate, Betaine, Vitamin B12, Life management

↑, increased; ↓, decreased; −, normal; C3, propionylcarnitine; cbl, cobalamin; CBS, cystathionine β-synthase; Mca, methylcitric acid; Met, methionine; Mma, methylmalonic acid; MTHFR, methylene-tetrahydrofolate reductase; MUT, methylmalonic-CoA mutase; Hcy, homocysteine; PCC, propionyl-CoA carboxylase.

In the long-term management of diagnosed patients, the effectiveness of treatment is evaluated by monitoring changes in metabolite levels and improvements in clinical manifestations, allowing for the adjustment in medication dosage, identification of metabolic decompensation, and prevention of complications (10). Metabolic examinations during follow-up also encompass the measurement of metabolites in DBS, urine, and serum. However, this process requires the collection of multiple biological samples and the utilization of different analytical methods, resulting in significant inconvenience. Neonates may undergo venous blood collection via femoral or external jugular vein puncture, which is more invasive than peripheral-blood testing. Urine metabolite detection using gas chromatography-mass spectrography (GC–MS) entails complex sample pretreatment and is time-consuming. Moreover, urinary metabolites are no longer reliable markers in patients with renal insufficiency (13). The utilization of liquid chromatography–tandem mass spectrometry (LC–MS/MS) for detecting specific biomarkers in DBS instead of additional testing for specific markers in serum and urine can address these issues, as reported in the two-tier NBS for targeted inherited metabolic disorders (14–16). However, data on the application of this method in the follow-up monitoring of inherited metabolic diseases are still insufficient (13, 17).

In this study, we developed an LC–MS/MS assay for quantifying three specific markers (methylmalonic acid, methylcitric acid, and homocysteine) in DBS, and further explored the clinical value of these DBS metabolites in evaluating post-treatment outcomes and monitoring follow-up progress in 228 patients diagnosed with MMA, PA, and homocysteinemia.

## 2 Materials and methods

### 2.1 Subjects and samples

This study enrolled 228 patients with inherited metabolic disorders, who were followed up after treatment in Peking University First Hospital between February 2017 and February 2018. The age of the patients ranged from 1 to 348 months (median age: 36 months), and the follow-up time ranged from 1.0 to 144.0 months (median time: 24.5 months). The cohort comprised 163 patients with combined MMA (cblC, *n* = 161; cblX, *n* = 1; cblJ, *n* = 1), 42 patients with isolated MMA (MUT deficiency, *n* = 41; cblA, *n* = 1), 17 patients with PA, and 6 patients with homocysteinemia (CBS deficiency, *n* = 2; MTHFR deficiency, *n* = 2; cblE, *n* = 1; cblG, *n* = 1). Basic demographic information of patients in each group was presented in Supplementary Table S1.

Personalized treatment was initiated upon diagnosis in all patients. The treatment strategies encompassed acute management, which included protein restriction, high-calorie intake, administration of L-carnitine and/or hydroxocobalamin, correction of metabolic acidosis and hyperammonemia, and symptomatic care, as well as long-term management. In patients with MMA, the long-term treatment comprised oral L-carnitine, intramuscular hydroxocobalamin for responsive MMA patients, betaine for combined MMA, natural protein restriction coupled with medical foods supplementation for isolated MMA, and treatment of complications. For patients with PA, daily management included oral L-carnitine, dietary intervention, and symptomatic treatment. Furthermore, long-term treatment for patients with homocysteinemia involved oral pyridoxine for CBS deficiency, supplementation with betaine and folic acid (1, 3, 18, 19). Patients’ residual DBS samples were collected after routine tests for blood amino acids and acylcarnitines and stored at −20°C (15).

**Figure 1:**
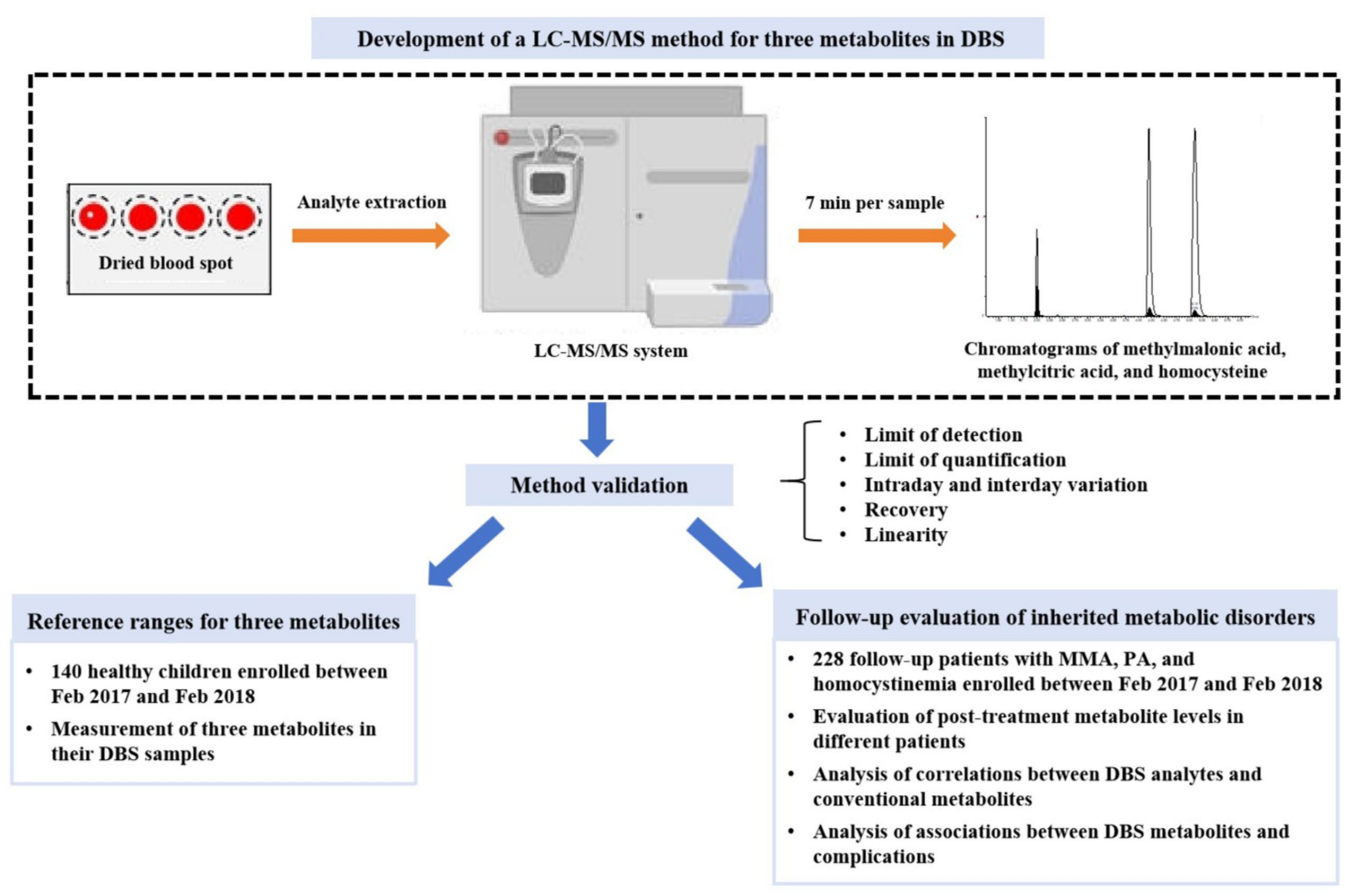
The flowchart of study design. DBS, dried blood spot; LC–MS/MS, liquid chromatography–tandem mass spectrometry; MMA, methylmalonic acidemia; PA, propionic acidemia.

To establish the reference ranges of methylmalonic acid, methylcitrate acid, and homocysteine in DBS, we recruited control subjects who underwent health examinations or NBS. The inclusion criteria were individuals under 18 years old, self-reported good health, and normal amino acid and acylcarnitine profiles. Exclusion criteria included confirmed diagnosis of inherited metabolic disorders, elevated serum homocysteine levels, and abnormal liver or renal function. A total of 140 healthy subjects (68 males and 72 females) with a median age of 7.5 months (range: 0.2–192 months) were enrolled. Residual DBS samples were collected after routine laboratory tests and stored at −20°C. The research design of this study was shown in Figure 1. This study was approved by Hospital Institutional Ethics Committee in accordance with the Declaration of Helsinki.

### 2.2 Routine metabolite analysis

Amino acids and acylcarnitines in DBS samples were detected using Waters ACQUITY TQD LC–MS/MS system (Waters, Milford, United States) and NeoBase Non-derivatized MSMS Kit (PerkinElmer, Turku, Finland). Data were collected and processed with Waters MassLynx Mass Spectrometry Software. Urinary organic acids were analyzed via GC–MS using a Shimadzu GCMS-QP2010 system (Shimadzu, Kyoto, Japan), and data were collected with GC–MS solution software. Serum homocysteine were determined using an ABBOTT I2000 chemiluminescence immunoassay analyzer (Abbott Laboratories, Illinois, United States).

### 2.3 Determination of three metabolites in DBS

Methylmalonic acid, methylcitric acid, and homocysteine in DBS were quantified using a modified LC–MS/MS method adapted from Turgeon et al. (20). Briefly, the analytes were extracted from a 3.0-mm DBS punch with the solution containing dithiothreitol and three isotope deuterium (d3) -labeled internal standards. The extracted compounds were dried, butylated to form butylesters, and separated chromatographically on a waters BEH C18 column (2.1 mm × 50 mm, 1.8 μm, 100 Å) at 40°C. Gradient elution involved mobile phase A (aqueous 0.1% formic acid) and mobile phase B (acetonitrile:water:formic acid, 80:20:0.1%) at a flow rate of 500 μL/min. Detection was conducted on a LC–MS/MS system (Waters ACQUITY UPLC-TQD, Milford, United States) in positive ion mode (5,000 V, 350°C), optimized to monitor the transitions of m/z 231 to 119 and m/z 234 to 122 for methylmalonic acid and d3-methylmalonic acid, m/z 375 to 199 and m/z 378.2 to 202.2 for methylcitric acid and d3-methylcitric acid, and m/z 192 to 118.1 and m/z 196 to 94 for homocysteine and d8-homocysteine.

The method was validated for limit of detection, limit of quantification, intraday variation, interday variation, recovery, and linearity. The linearity of standard curve was assessed across a concentration range of 0–500 μmol/L.

### 2.4 Statistical analysis

Normality of data was tested using Shapiro–Wilk test. Nonparametric variables such as DBS and urine methylmalonic acid, DBS and urine methylcitric acid, and DBS and serum homocysteine were presented as median (interquartile range). Differences of metabolite levels in patients with different inherited metabolic diseases were compared by independent-sample Kruskal-Wallis test with Bonferroni correction. Comparisons between patients with different disease severity were performed using Mann–Whitney U test (age, treatment duration, and DBS metabolite) and chi-square test (gender), respectively. Correlations between DBS analytes and urine or serum metabolites were tested by calculating the Spearman rank correlation coefficient. The 95% reference interval was estimated based on the Clinical and Laboratory Standards Institute (CLSI) guideline (21). Statistical analysis was performed using SPSS 20.0 (IBM, Armonk, United States), with significance set at *p* < 0.05. Reference ranges were calculated in MedCalc 20.0 (MedCalc Software, Ostend, Belgium).

## 3 Results

### 3.1 Method validation

**Figure 2:**
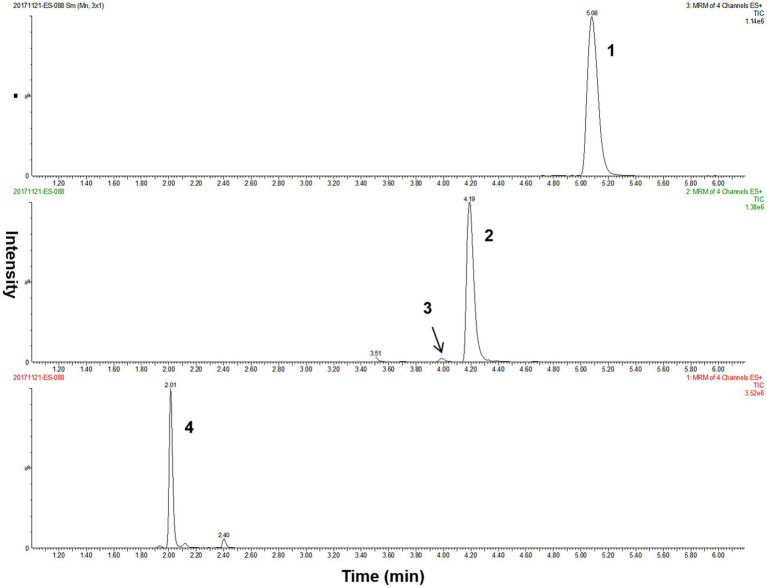
The LC–MS/MS chromatograms of analytes from a quality control DBS sample. Peak 1, methylcitric acid; peak 2, methylmalonic acid; peak 3, succinic acid; peak 4, homocysteine. DBS, dried blood spot; LC–MS/MS, liquid chromatography–tandem mass spectrometry.

The overall performance of our LC–MS/MS method for detecting methylmalonic acid, methylcitric acid, and homocysteine in DBS was summarized in Table 2. The differentiation between methylmalonic acid and its interfering substance, succinic acid (isobaric with methylmalonic acid), was achieved through distinct retention times (4.2 min for methylmalonic acid and 4.0 min for succinic acid) (Figure 2). The method exhibited strong linearity, with *R*^2^ coefficients of 0.995 for methylmalonic acid, 0.996 for methylcitric acid, and 0.995 for homocysteine. Intra- and interday variations were within the ranges of 1.0–9.9% and 3.1–9.9%, and recoveries ranged from 84 to 101% as detailed in Table 2.

**Table 2:** Assay performance of the LC–MS/MS method for methylmalonic acid, methylcitric acid and homocysteine in dried blood spots.

	Methylmalonic acid	Methylcitric acid	Homocysteine
Retention time (min)	4.2	5.1	2.0
Limit of detection (μM)	0.076	0.039	0.041
Limit of quantification (μM)	0.32	0.16	0.32
Intraday variation (%)	1.6–9.2	1.6–9.9	1.0–5.9
Interday variation (%)	4.9–9.9	3.7–8.8	3.1–6.4
Linearity (*R*^2^)	0.995	0.996	0.995
Recovery (%)	93–101%	92–101%	84–91%
Reference interval (μmol/L, 2.5th to 97.5th)	0.04–1.02	0.02–0.27	1.05–8.22

Limit of detection was defined as mean plus 3 standard deviations of blank, and limit of quantification was defined as mean plus 10 standard deviations of blank. Intra- and inter-day variations were evaluated by the analysis of quality control samples at three levels (*n* = 6). LC–MS/MS, liquid chromatography–tandem mass spectrometry.

### 3.2 Reference ranges for three metabolites in DBS

In DBS samples from healthy controls, the average concentrations of methylmalonic acid, methylcitric acid, and homocysteine were 0.43 μmol/L (interquartile: 0.31–0.55 μmol/L), 0.10 μmol/L (interquartile: 0.07–0.14 μmol/L), and 3.90 μmol/L (interquartile: 2.66–5.07 μmol/L), respectively. The data for these DBS metabolites exhibited a positively skewed distribution based on the Shapiro–Wilk test (*p* < 0.001 for methylmalonic acid, *p* < 0.001 for methylcitric acid, and *p* = 0.041 for homocysteine). Turkey method was used to remove outliers, but data were still non-parametric distribution. Therefore, the 95% reference interval was calculated according to the percentile method recommended by the CLSI guideline (21). The normal reference ranges (2.5th–97.5th percentile) were estimated as 0.04–1.02 μmol/L for DBS methylmalonic acid, 0.02–0.27 μmol/L for DBS methylcitric acid, and 1.05–8.22 μmol/L for DBS homocysteine.

### 3.3 Metabolite levels following treatment in different patients

**Figure 3:**
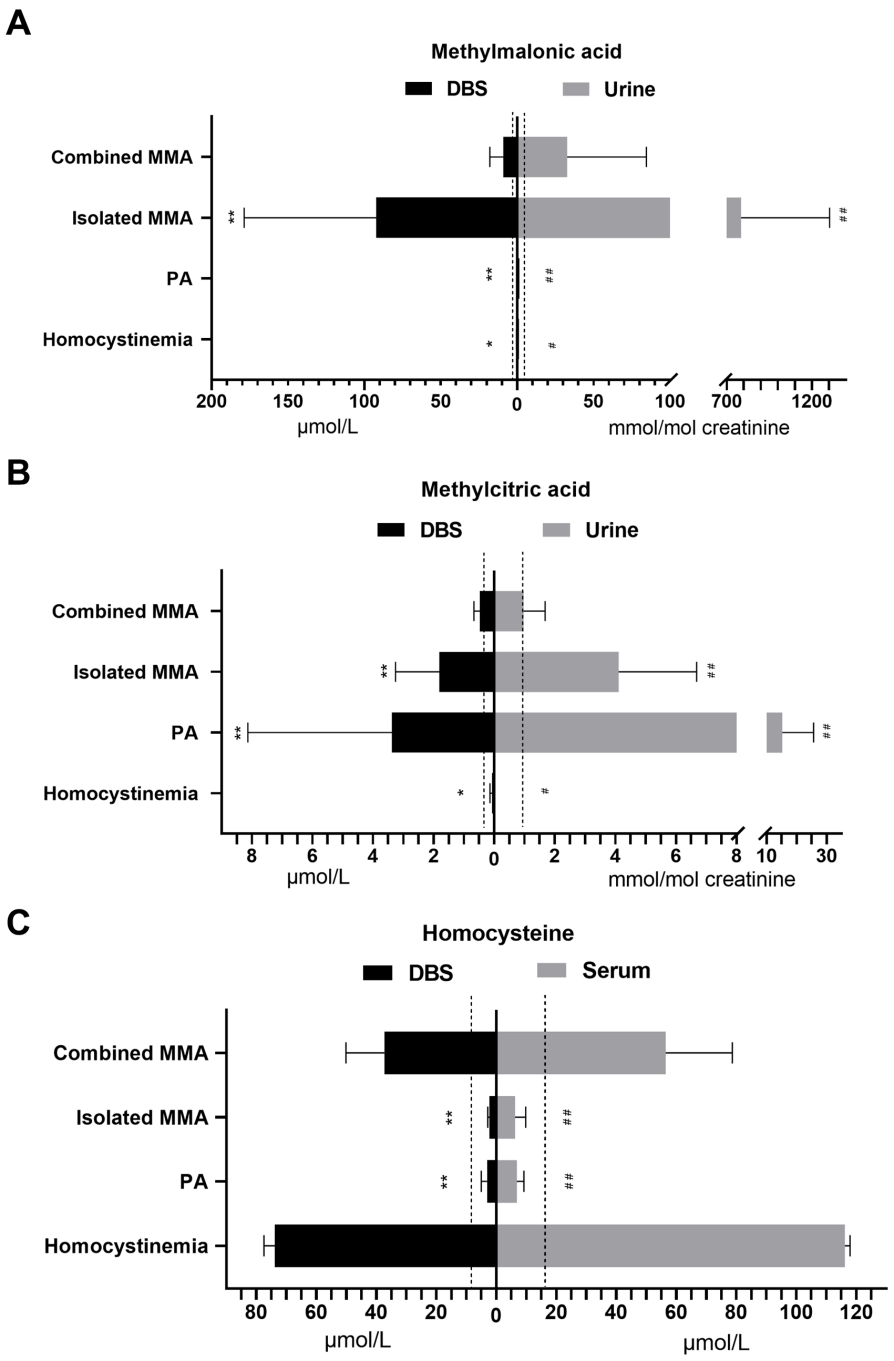
The concentrations of methylmalonic acid in DBS and urine **(A)**, methylcitric acid in DBS and urine **(B)**, and homocysteine in DBS and serum **(C)** from patients with different diseases. The dashed lines indicate the upper limit of reference ranges. Asterisks indicate significant differences in DBS metabolite levels compared with those in patients with combined MMA (^*^p < 0.05, ^**^p < 0.001). Pound signs indicate significant differences in urine metabolite levels compared with those in patients with combined MMA (^#^*p* < 0.05, ^##^*p* < 0.001). DBS, dried blood spot; MMA, methylmalonic acidemia; PA, propionic acidemia.

In our study of 228 patients receiving therapy, the analysis of metabolite concentrations revealed characteristic biochemical patterns in the majority of patients (Table 3; Figure 3). Among patients with combined MMA, elevated levels of DBS methylmalonic acid, methylcitric acid, and homocysteine were observed in 99.4, 81.6, and 100% of cases, respectively. Similarly, elevated levels of urine methylmalonic acid, methylcitric acid, and serum homocysteine were found in 94.5, 48.5, and 100% of patients with combined MMA, respectively. Among the 42 patients with isolated MMA, all exhibited increased concentrations of DBS and urine methylmalonic acid, while 95.2 and 88.1% of these patients displayed increased levels of DBS and urine methylcitric acid, respectively. Furthermore, all patients with PA demonstrated elevated levels of DBS and urine methylcitric acid, while patients with homocystinemia exhibited increased concentrations of DBS and serum homocysteine.

**Table 3:** Metabolite concentrations in samples from different groups of patients.

	DBS (μmol/L)					Urine (mmol/mol creatinine)		Serum (μmol/L)
	Met	C3	Mma	Mca	Hcy	Mma	Mca	Hcy
Reference range	10.00–50.00^a^	1.00–4.00^a^	0.04–1.02^b^	0.02–0.27^b^	1.05–8.22^b^	0.20–3.60^a^	0.00–1.00^a^	6.00–17.00^a^
Combined methylmalonic acidemia (*n* = 163)	18.20 (14.90–23.20)	5.67 (4.18–7.99)	9.21 (5.09–17.91)	0.47 (0.25–0.67)	37.33 (28.04–50.10)	32.76 (12.56–84.59)	0.97 (0.48–1.68)	56.50 (45.65–78.70)
Isolated methylmalonic acidemia (*n* = 42)	19.85 (17.10–25.43)	22.55 (13.69–34.20)	92.15 (39.80–178.78)	1.81 (0.94–3.26)	2.24 (1.78–2.82)	784.21 (455.32–1303.51)	4.11 (1.86–6.68)	6.27 (3.06–9.77)
Propionic acidemia (*n* = 17)	17.90 (14.80–19.70)	35.81 (24.46–38.90)	0.25 (0.15–0.41)	3.38 (2.41–8.13)	3.08 (2.01–5.06)	0	15.30 (11.83–25.70)	6.85 (5.77–9.20)
Homocysteinemia (*n* = 6)	16.35 (13.02–135.48)	1.23 (1.01–1.78)	0.36 (0.25–0.56)	0.08 (0.04–0.14)	73.87 (52.43–77.49)	0	0	116.15 (99.55–117.90)

C3, propionylcarnitine; DBS, dried blood spot; Mca, methylcitric acid; Met, methionine; Mma, methylmalonic acid; Hcy, homocysteine.

Data are presented as median (25th–75th percentile).

a Reference ranges were well established and used for routine clinical testing.

b Reference ranges were based on the data of dried blood spots from 140 healthy controls in this study.

The levels of DBS and urine methylmalonic acid in isolated MMA patients were significantly higher than those in combined MMA patients (both *p* < 0.001), as indicated in Figure 3A. Patients with PA had higher concentrations of both DBS and urine methylcitric acid (*p* < 0.001) compared to patients with combined or isolated MMA (Figure 3B). Both patients with combined MMA and those with homocystinemia showed significantly elevated homocysteine levels compared to the upper limit of normal ranges, with higher average levels in serum than in DBS samples (*p* < 0.001, Figure 3C).

### 3.4 Correlations between DBS analytes and conventional metabolites

**Figure 4:**
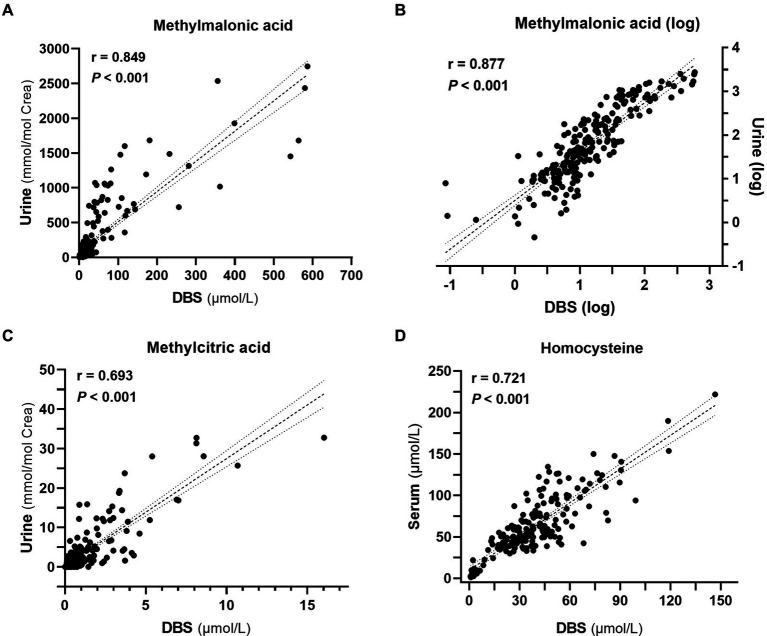
Correlation analyses among metabolite levels (*n* = 228). **(A)** Correlation between methylmalonic acid levels in dried blood spots (DBS) and urine. **(B)** Correlation between the logarithm-transformed methylmalonic acid levels in DBS and urine. **(C)** Correlation between methylcitric acid levels in DBS and urine. **(D)** Correlation between homocysteine levels in DBS and serum.

In the analysis of metabolite levels in DBS, urine, and serum samples from 228 patients, we found a robust positive correlation between DBS and urine methylmalonic acid (correlation coefficient *r* = 0.849, *p* < 0.001, Figure 4A), which was further improved with logarithmized data (*r* = 0.877, *p* < 0.001, Figure 4B). Additionally, favorable positive correlations were also observed between DBS and urine methylcitric acid (*r* = 0.693, *p* < 0.001, Figure 4C), as well as between DBS and serum homocysteine (*r* = 0.721, *p* < 0.001, Figure 4D).

**Figure 5:**
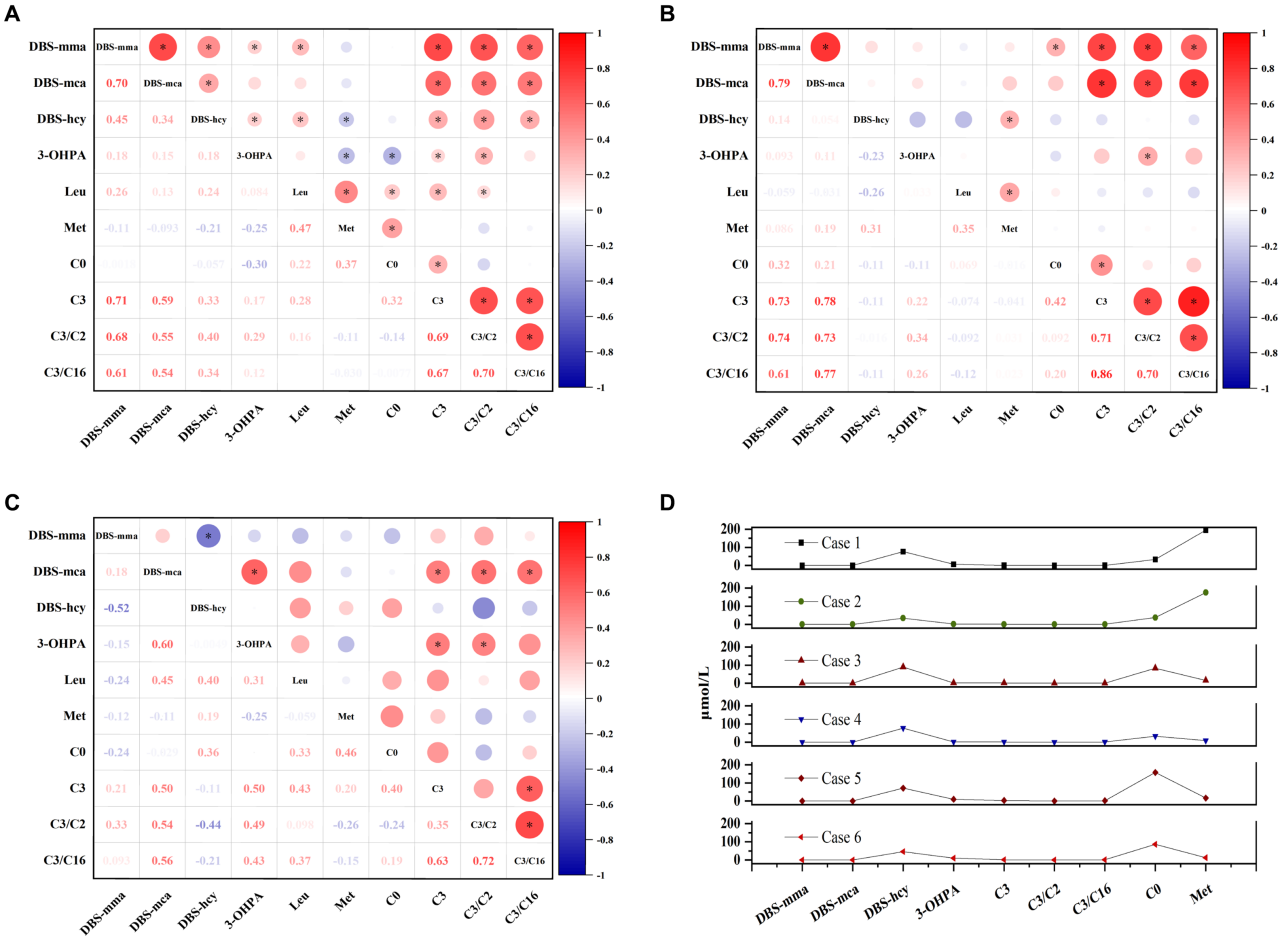
The associations among DBS indicators and other conventional metabolites in patients with combined methylmalonic acidemia **(A)**, isolated methylmalonic acidemia **(B)**, propionic acidemia **(C)**, and homocysteinemia **(D)**, respectively. Red values indicate a positive correlation and blue values indicate a negative correlation. The bubble size reflects the magnitude of correlation, and the asterisk represents *p* < 0.05. C0, free carnitine; C2, acetylcarnitine; C3, propionylcarnitine; C16, palmitoylcarnitine; DBS, dried blood spot; hcy, homocysteine; Leu, leucine; mca, methylcitric acid; Met, methionine; mma, methylmalonic acid; 3-OHPA, 3-hydroxypropionic acid.

We further analyzed the correlation between DBS indicators and other conventional metabolites. In patients with combined MMA, DBS methylmalonic acid and methylcitrate exhibited strong positive correlations with propionylcarnitine and its ratios, whereas DBS homocysteine showed weaker correlations with essential amino acids (leucine and methionine) and propionylcarnitine (Figure 5A). Elevated levels of DBS methylmalonic acid and methylcitrate in patients with isolated MMA were significantly correlated with propionylcarnitine and its ratios (Figure 5B). Additionally, in PA patients, the markedly increased DBS methylcitrate showed positive correlations with 3-hydroxypropionic acid and propionylcarnitine (Figure 5C). Due to the limited number of patients with homocysteinemia, accurate analysis of metabolite correlations was challenging. Notably, elevated levels of both homocysteine and methionine were observed in two patients with CBS deficiency, while the remaining four patients with homocysteine remethylation disorder had normal methionine levels, consistent with the disease’s biochemical phenotype (Figure 5D).

### 3.5 Association between post-treatment metabolites and complications

DBS metabolites were regularly monitored over the past year, revealing that the majority exhibited decreased or stable levels following treatment. However, a small subset of patients (5 cases of MMA, 3 cases of PA, and 1 case of homocysteinemia), particularly those with severe symptoms or unstable conditions, showed fluctuating or significantly elevated metabolite levels (Supplementary Figure S1).

**Figure 6:**
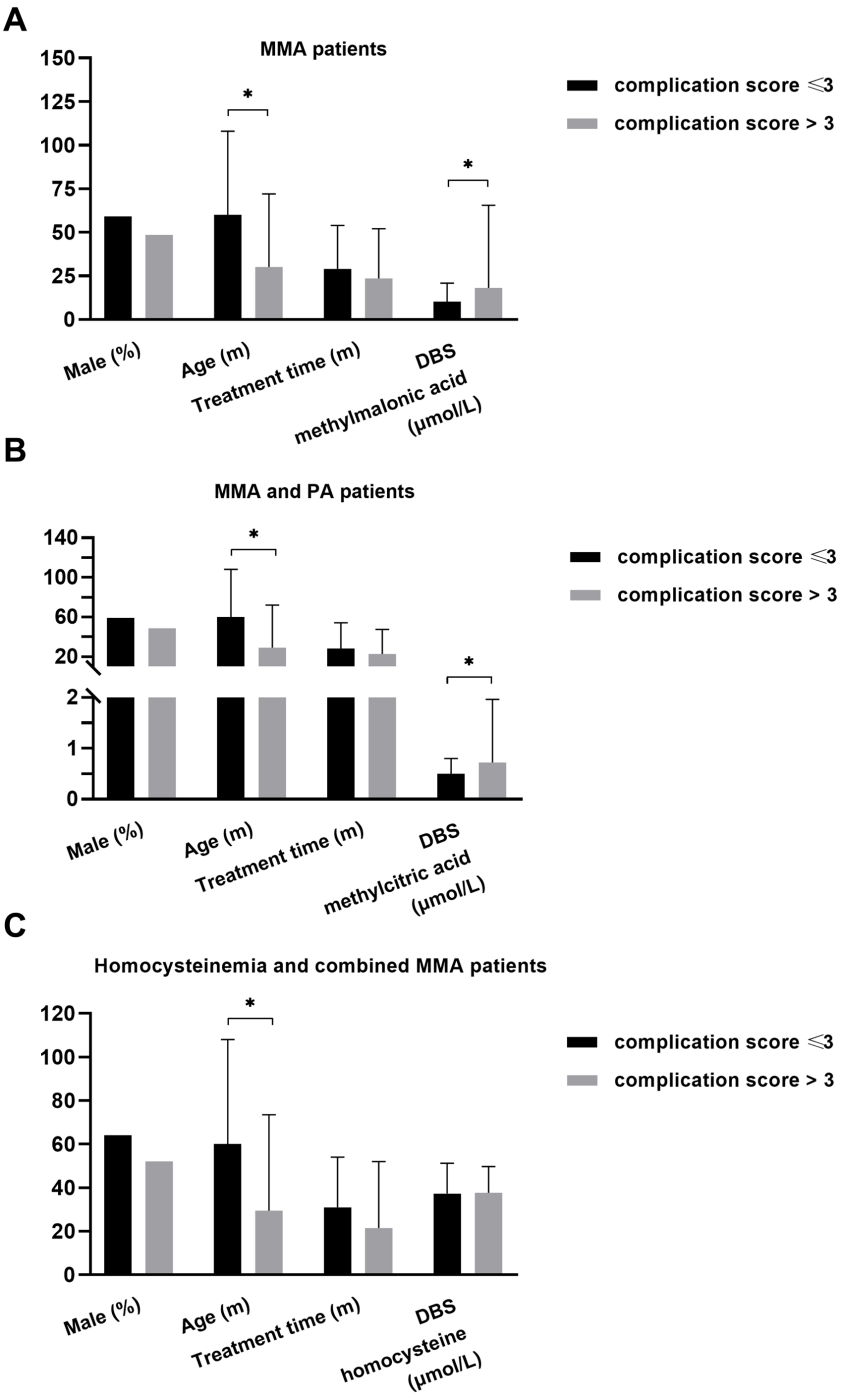
The relationship of DBS metabolites with disease burden in different patients. **(A)** Younger age (*p* = 0.016) and higher level of DBS methylmalonic acid (*p* = 0.007) were observed in MMA patients with higher cumulative complication score. **(B)** Younger age (*p* = 0.005) and higher level of DBS methylcitric acid (*p* = 0.004) were observed in MMA and PA patients with higher complication score. **(C)** Younger age (*p* = 0.019) was associated with higher complication score in individuals with homocysteinemia and those with combined MMA *p < 0.05. DBS, dried blood spot; MMA, methylmalonic acidemia; PA, propionic acidemia.

Subsequently, follow-up data from 200 patients were collected, documenting the type of onset (early onset), aberrations in growth, mental and motor development, vision, hearing, kidney function, heart health, blood parameters, digestive system, and bone health, as well as the frequent occurrence of metabolic decompensation (≥3) in the past year. Each identified abnormality was scored as 1, contributing to the cumulative complication score calculation. We then compared the DBS metabolite levels between patients with complication scores ≤3 and >3, as depicted in Figure 6.

The data from patients with combined MMA and isolated MMA were analyzed to investigate the association between DBS methylmalonic acid and complications (Figure 6A). The results revealed that patients with a complication score > 3 were younger (*p* = 0.016) and exhibited higher levels of DBS methylmalonic acid (*p* = 0.007) compared to those with a score ≤ 3. No significant differences in gender or treatment duration were observed between the two groups. Among patients with combined MMA, isolated MMA, and PA, individuals with a complication score > 3 also showed younger age (*p* = 0.005) and higher average level of DBS methylcitric acid (*p* = 0.004) compared to those with a score < 3. However, there were no significant differences in terms of gender or treatment duration (Figure 6B). Additionally, age was associated with complication score (*p* = 0.019) in patients with combined MMA and those with homocysteinemia, while other variables such as DBS homocysteine, gender, and treatment duration were not significantly related to complication score (Figure 6C).

## 4 Discussion

This study demonstrated favorable assay performance of the LC–MS/MS method for the three DBS parameters, methylmalonic acid, methylcitric acid, and homocysteine, with intra- and inter-precision of less than 10%, recoveries of 84–101%, and good linearity (*R*^2^ 0.995–0.996), comparable to reported LC–MS/MS methods (16, 20, 22–26), which mainly aimed to improve the detection rate of target disorders and reduce the rate of false positivity in NBS. However, given that LC–MS/MS methodologies are predominantly custom-tailored by individual laboratories, discrepancies often arise in the measurement of identical analytes across different settings. Such variability primarily stems from diverse sample preparation methods, chromatographic separation parameters, mass spectrometry settings, and ionization efficiencies, all of which can compromise the reproducibility and comparability of results among laboratories. Consequently, it is necessary to implement standardized protocols and rigorous quality control measures to safeguard the consistency and credibility of analytical outcomes (27, 28). Looking ahead, integrating these DBS markers into an external quality assessment (EQA) scheme will further enhance accuracy and comparability of results among laboratories.

Reference ranges suggested by Wang et al. (13)for methylmalonic acid, methylcitric acid, and homocysteine in DBS samples from children in Nangjing, China were 0.04–2.37 μmol/L, 0.01–0.65 μmol/L, and 0.65–7.73 μmol/L, respectively. Al-Dirbashi et al. (24) found the median concentration of methylcitric acid to be 0.06 μmol/L (range: 0–0.63 μmol/L) in neonatal DBSs which yielded normal NBS results. In another study, the reference range for homocysteine was estimated as 5.4–10.7 μmol/L based on the analysis of 99 neonatal DBS samples (29). In our study, the estimated reference intervals for methylmalonic acid, methylcitric acid, and homocysteine were 0.04–1.02 μmol/L, 0.02–0.27 μmol/L, and 1.05–8.22 μmol/L, which were comparable with previous findings. However, it should be noted that, in addition to certain inherited metabolic disorders, deficiencies in vitamin B12 and folate secondary to dietary restriction or malabsorption, can also lead to mild increases in DBS methylmalonic acid, methylcitric acid, and homocysteine (25, 30, 31). Furthermore, age may also be a factor affecting DBS metabolite levels. It was reported that infants younger than 6 months had higher concentrations of circulating methylmalonic acid and homocysteine than older children (32). The concentration of DBS methylmalonic acid in newborns was approximately six times higher than that in healthy young adult women, which was potentially related to the reduced physiological renal clearance in infants (22). However, another study involving approximately 2,000 healthy infants found that the concentrations of methylmalonic acid, methylcitric acid, and homocysteine in DBS were not affected by gender, age, birth weight, or gestational age (33). Therefore, future research should further elucidate the levels of DBS metabolites across different age groups, genders, and ethnicities, and establish reference intervals for these diverse subgroups, which will aid in better identifying patients with vitamin B12 deficiency and mild type of congenital disorders of cobalamin metabolism.

For individuals with MMA and PA, conventional long-term therapies such as dietary protein restriction and supplementation of carnitine and vitamins may not effectively prevent the recurrence of metabolic decompensation or chronic long-term complications like mental retardation, impaired vision, kidney disease, cardiomyopathy, and prolonged QT interval (1, 17). Regular monitoring of metabolic parameters is essential for timely adjustments to treatment plans and follow-up strategies (3, 10). Routine metabolic assessments for MMA patients, particularly those combined with homocysteinemia, typically include DBS propionylcarnitine analysis by LC–MS/MS, urine methylmalonic acid measurement by GC–MS, and serum homocysteine testing by immunoassay. For the PA patients, monitoring DBS propionylcarnitine via LC–MS/MS and urine methylcitric acid using GC–MS are crucial follow-up indicators. Additionally, patients with homocystinemia require regular evaluation of serum homocysteine and/or DBS methionine. The utilization of LC–MS/MS to detect disease-specific markers like methylmalonic acid, methylcitric acid, and homocysteine in DBS offers advantages in terms of convenient sample collection and reduced detection time, facilitating the biochemical diagnosis of target disorders (14, 15, 26). However, further assessment is needed to determine its clinical utility for patient monitoring during follow-up.

The present study assessed the clinical value of three DBS biomarkers (methylmalonic acid, methylcitric acid, and homocysteine) in evaluating therapeutic response and disease progression in a large cohort of cases. Our findings showed that only a small number of patients achieved normal metabolite levels following standard therapy, with the majority maintaining typical biochemical phenotypes during follow-up. Elevated DBS methylmalonic acid levels were observed exclusively in MMA patients, particularly those with isolated MMA. And PA patients demonstrated significantly higher average concentrations of DBS methylcitric acid compared to MMA patients. Moreover, the concentrations of these three biomarkers in DBS were parallel to those in urine or serum, though the values in urine or serum were consistently higher than those in DBS, consistent with existing literatures (13, 17). Notably, our study revealed an association between DBS metabolite levels and disease burden: MMA patients with more severe phenotype and more complications exhibited higher DBS methylmalonic acid levels, while patients with more severe MMA and PA also displayed higher concentrations of DBS methylcitric acid. Similarly, in a cohort of 538 patients with combined MMA, traditional metabolic markers such as propionylcarnitine ratio, methylmalonic acid, methylcitric acid, and homocysteine were higher in the poor prognosis group, characterized by varying degrees of physical and mental impairment (34). Another study indicated that higher levels of biomarkers like propionylcarnitine and methylcitrate were linked to a more severe intellectual disability profile of PA (35). Maines et al. (17) also observed that plasma methylcitric acid mirrored disease burden, although the specific clinical parameters used to assess disease burden differed from those in our study. It is important to note that there is currently no validated clinical scoring system for assessing disease severity in MMA or PA patients (17, 36, 37). In addition, we observed that younger age was associated with higher complication score. Since there was no difference in treatment duration between patient groups, this association can be explained as a correlation between onset age and disease burden, that is, earlier age at onset was related to more severe disease. Previous studies indicated that the age of onset was one of the important factors affecting prognosis (10, 12). Early-onset patients had a higher mortality rate, while late-onset patients had a milder phenotype and may have a better prognosis (10, 38). In clinical practice, biomarkers capable of predicting disease severity and multisystem involvement are needed to help stratify patients and monitor disease progression. Future longitudinal studies can further clarify the ability of DBS metabolites to predict long-term prognosis of inherited metabolic disorders. Moreover, subsequent studies may also explore the value of potential biomarker panel that includes additional indicators of disease burden, such as FGF21 (39), for assessing disease severity or monitoring specific complications.

In the cohort of 228 patients with inherited metabolic disorders, we also observed robust correlations among these three DBS makers and conventional metabolic parameters utilized for disease monitoring. Notably, MMA stands as the most prevalent organic acidemia in China, with approximately 70% of patients exhibiting combined MMA with homocysteinemia (18, 40). For individuals diagnosed with combined MMA, routine monitoring of methylmalonic acid and homocysteine levels is imperative to facilitate timely adjustments to treatment strategies. Our findings unveiled strong correlations between DBS and urine methylmalonic acid (*r* = 0.849), and between DBS and serum homocysteine (*r* = 0.721), consistent with the findings of a prior study conducted by Wang et al. involving 50 MMA and PA patients during follow-up (13). Despite the strong correlation observed between DBS methylmalonic acid and classical biomarkers propionylcarnitine and its ratio, methylmalonic acid was considered to have superior specificity and stability compared to propionylcarnitine, remaining unaffected by stressors such as infection (13, 20). Consequently, the utilization of DBS methylmalonic acid and homocysteine may be extended to encompass follow-up monitoring of MMA patients. Furthermore, methylcitrate and 3-hydroxypropionic acid are recognized for their heightened specificity in PA diagnosis relative to propionylcarnitine (41). In our current investigation, significant associations were observed among DBS methylcitrate, propionylcarnitine, and 3-hydroxypropionic acid in follow-up PA patients, with a notable linear correlation between DBS and urine methylcitrate. Previous study suggested that DBS methylcitric acid may serve as a more stable biomarker compared to 3-hydroxypropionic acid and perform better in PA diagnosis (23). Therefore, these findings indicate the promising potential of DBS methylcitric acid as an alternative marker to traditional metabolic parameters in monitoring PA patients. Collectively, the LC–MS/MS assay developed in this study is capable of simultaneously quantifying three metabolic biomarkers in DBS, offering the benefits of high detection efficiency, reliability of its analytical capacity, and simplicity and minimal invasiveness of sample collection. This method holds promising potential for post-treatment monitoring and disease progression evaluation in select inherited metabolic disorders. Nonetheless, the necessity for additional equipment acquisition may present a financial challenge for some healthcare facilities. Given the extensibility of the LC–MS/MS approach, there is a prospect for the development of expanded detection panels encompassing a broader array of disease markers to assist in the diagnosis and monitoring of a wider spectrum of inherited metabolic conditions, thereby optimizing cost-effectiveness.

We acknowledge several limitations in our study. Firstly, the reference ranges of DBS metabolites in this study were established based on single-center samples, and the effect of vitamin B12 and folate levels on metabolite concentrations was not ruled out. Secondly, the complete laboratory and clinical data of some patients were unavailable, resulting in their exclusion from the analysis of the association between metabolites and disease burden. Thirdly, samples were collected only from patients in a stable metabolic state, precluding a comparison of biomarker levels between metabolic decompensation and metabolic homeostasis. Fourthly, post-transplantation samples from certain patients were not accessible, precluding an analysis of changes in biomarker levels before and after transplantation.

## 5 Conclusion

The LC–MS/MS method developed in this study demonstrated robust capability in detecting methylmalonic acid, methylcitric acid, and homocysteine in DBS. The successful detection of these three biomarkers in DBS aligned with their detection profiles in urine or serum, suggesting the feasibility of utilizing this method for longitudinal monitoring in patients with MMA, PA, and homocysteinemia. However, elucidating the precise relationship between these DBS metabolites and disease burden warrants additional investigation.

## Data availability statement

The raw data supporting the conclusions of this article will be made available by the authors, without undue reservation.

## Ethics statement

The studies involving humans were approved by Clinical Research Ethics Committee of Peking University First Hospital. The studies were conducted in accordance with the local legislation and institutional requirements. The human samples used in this study were acquired from the residual samples in the laboratory after routine clinical testing. Written informed consent for participation was not required from the participants or the participants’ legal guardians/next of kin in accordance with the national legislation and institutional requirements.

## Author contributions

YL: Data curation, Formal analysis, Writing – original draft, Writing – review & editing. XM: Data curation, Formal analysis, Writing – review & editing. LK: Data curation, Project administration, Writing – review & editing. YJ: Investigation, Methodology, Writing – review & editing. ML: Investigation, Methodology, Writing – review & editing. JS: Investigation, Methodology, Writing – review & editing. HL: Methodology, Writing – review & editing. YC: Conceptualization, Writing – review & editing. YY: Conceptualization, Project administration, Writing – review & editing.
