# Supplementary material for: The utility of methylmalonic acid, methylcitrate acid, and homocysteine in dried blood spots for therapeutic monitoring of three inherited metabolic diseases
Source: Front Nutr. 2024 Jun 20;11:1414681. doi: 10.3389/fnut.2024.1414681 (PMC11222987; doi:10.3389/fnut.2024.1414681)
Supplement: Supplementary file 1 [file Table_1.DOCX]

## Supplementary Table 1 Demographic data and disease burden of different disease groups

|  | Combined methylmalonic acidemia  (n = 163) | Isolated methylmalonic acidemia  (n = 42) | Propionic acidemia  (n = 17) | Homocysteinemia  (n = 6) |
| --- | --- | --- | --- | --- |
| Gender (male) | 98, 60.1% | 15, 35.7% | 8, 47.1% | 4, 66.7% |
| Age (months) | 48.0 (12.0 - 100.0) | 36.0 (16.5 - 72.0) | 24.0 (8.0 - 30.0) | 15.5 (4.8 - 26.3) |
| Treatment duration (months) | 29.0 (10.0 - 54.0 ) | 27.0 (12.3 - 60.0) | 8.0 (4.0 - 42.0) | 14.5 (3.8 - 24.5) |
| Complication score^*^ | 3.0 (2.0 - 4.0) | 4.0 (3.0 - 5.0) | 3.0 (2.0 - 4.0) | 4 (3.3 - 4.8) |
| Causing gene | *MMACHC* (n = 161)  *ABCD4* (n = 1)  *HCFC1* (n = 1) | *MMUT* (n = 41)  *MMAA* (n = 1) | *PCCA* (n = 7)  *PCCB* (n = 10) | *CBS* (n = 2)  *MTHFR* (n = 2)  *MTRR* (n = 1)  *MTR* (n = 1) |

^*^Complication score is calculated by early onset (neonatal or infantile onset), aberrations in growth, mental and motor development, vision, hearing, kidney function, heart health, hematologic system, digestive system, and bone health. Data are shown as median and interquartile or number and percentage.
