# Supplementary material for: The utility of methylmalonic acid, methylcitrate acid, and homocysteine in dried blood spots for therapeutic monitoring of three inherited metabolic diseases
Source: Front Nutr. 2024 Jun 20;11:1414681. doi: 10.3389/fnut.2024.1414681 (PMC11222987; doi:10.3389/fnut.2024.1414681)
Supplement: Supplementary file 2 [file Image_1.pdf]

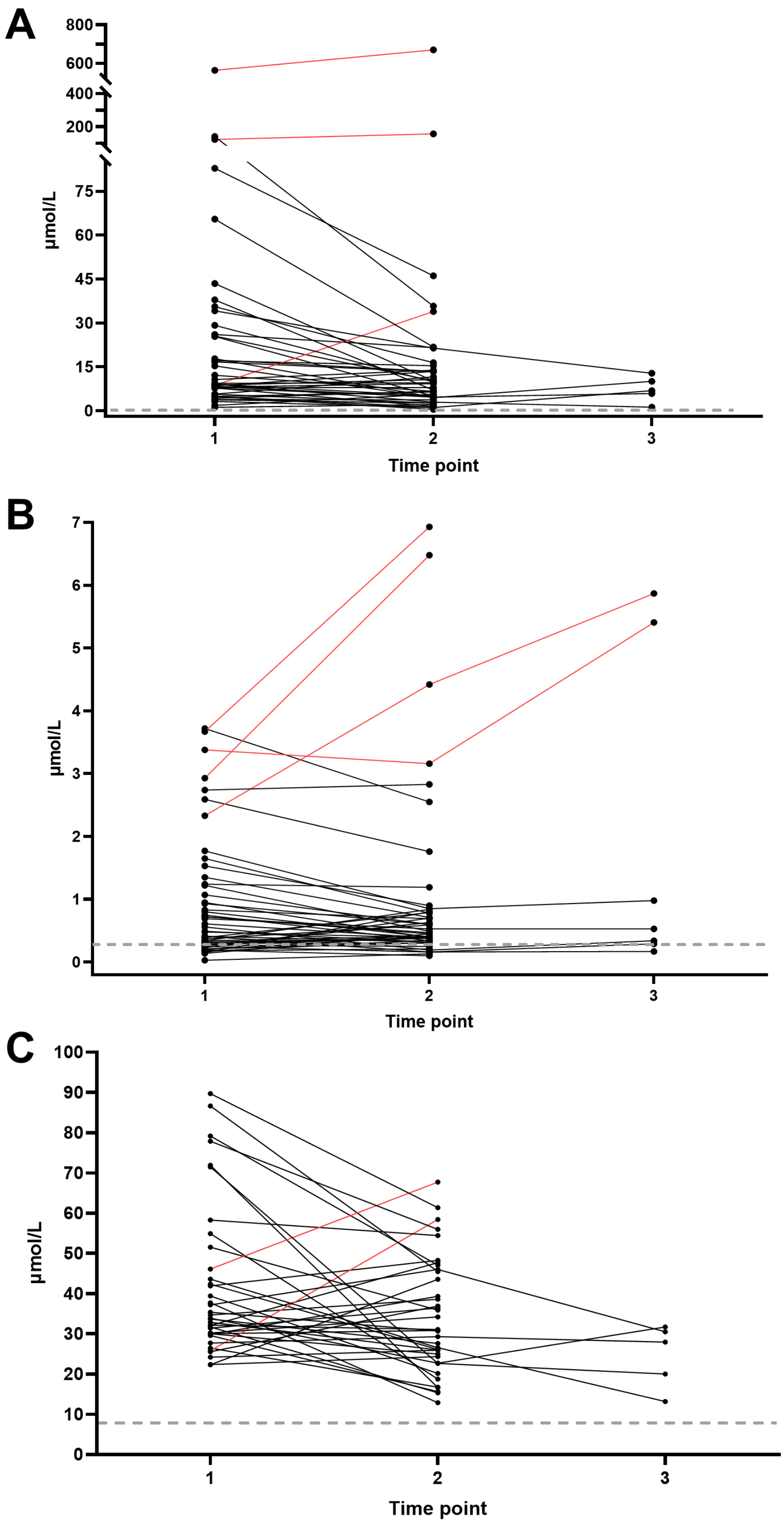
A
800
600
400
200
75
60
45
30
15
0
µmol/L
1
2
3
Time point
B
7
6
5
4
3
2
1
0
µmol/L
1
2
3
Time point
C
100
90
80
70
60
50
40
30
20
10
0
µmol/L
1
2
3
Time point

Supplementary Figure 1. The post-treatment levels of DBS methylmalonic acid **(A)**, methylcitric acid **(B)**, and homocysteine **(C)** over the course of the past year, with detection intervals occurring every 3-6 months. **(A)** The red lines indicate that three cases of combined or isolated MMA had elevated levels of methylmalonic acid, while these patients remained clinically stable. **(B)** The red lines highlight sharply increased levels of methylcitrate among three patients with PA (experiencing severe symptoms or episodes of metabolic decompensation) and one patient with isolated MMA (remaining stable). **(C)** The red lines indicate markedly elevated homocysteine levels detected in one patient with combined MMA (in stable condition) and one patient with homocysteinemia (after ventriculoperitoneal shunt surgery). DBS, dried blood spot; MMA, methylmalonic acidemia; PA, propionic acidemia.
